# Relationship between parental locus of control and caries experience in preschool children – cross-sectional survey

**DOI:** 10.1186/1471-2458-8-208

**Published:** 2008-06-12

**Authors:** Erika Lenčová, Hynek Pikhart, Zdeněk Broukal, Georgios Tsakos

**Affiliations:** 1 Institute of Dental Research – 1st Faculty of Medicine of the Charles University and General Teaching Hospital, Prague, the Czech Republic; 2 Department of Epidemiology and Public Health, University College London, London, UK

## Abstract

**Background:**

Due to high prevalence and serious impacts, childhood caries represents a public health issue. Behavioural risk factors such as locus of health control have been implicated in the development of the disease; however their association with childhood caries has not been thoroughly studied. The aim of this cross-sectional survey was to assess the relationship between parental locus of health control and caries experience and untreated caries of their preschool children in a representative sample in Czech Republic, adjusting for relevant sociodemographic characteristics.

**Methods:**

A representative sample of 285 preschool children and their parents was recruited. Study data included children's dental status recorded in nurseries and parental questionnaires with 13 attitudinal items regarding locus of control (LoC) in caries prevention. The association between parental locus of control and children's caries experience and level of untreated caries was analysed using logistic regression, adjusting for the effect of key sociodemographic variables.

**Results:**

There was a statistically highly significant linear trend between increased parental LoC and higher probability of the children to be free from untreated caries, independent from the effect of sociodemographic variables of children and parents. A similar highly statistically significant trend, although not entirely linear, and independent from sociodemographic variables was observed with respect to the chance of the children to be free from caries experience with increasing strength of parental LoC. After full adjustment, children in the strongest parental LoC quintile were 2.81 (1.23–6.42, p< 0.05) times more likely to be free from untreated caries in comparison to the weakest parental LoC quintile and 2.32 (1.02–5.25, p< 0.05) times more likely to be free from caries experience in comparison to the weakest parental LoC quintile.

**Conclusion:**

The findings support the hypothesis that higher internal parental LoC is associated with better control of both untreated caries and caries experience in their preschool children and highlight that a more internal LoC within the family is advantageous in the prevention of dental caries.

## Background

Childhood caries may result into severe impairment of both general and oral health [1,2]. Its prevalence in both developing and industrial countries is relatively high and ranges from 17% to 75% in different countries and populations [3-9], therefore it can be viewed as a relevant public health problem. Severe forms of childhood caries in primary dentition represent a symptom of other paediatric disorders and reflect lack of adequate care for children [10]. In this context, childhood caries should be addressed from the broader paediatric and public health rather than solely clinical dental standpoint, as viewing it as only a problem of dentists restricts the interest of the society to effectively solve it [11].

Dental caries aetiology is multifactorial, and the main aetiological factors include cariogenic bacteria, frequent intake of fermentable carbohydrates, disorders of salivary production and composition and poor mineralization of hard dental tissues [12]. The above-mentioned major risk factors interact, but still they do not always fully explain the distribution of the disease [13]. In an effort to further investigate complex interactions of the risk factors involved in the aetiology of childhood caries, research has focused on socioeconomic, psychological and behavioural risk factors as these could act as indirect causal agents [14]. With respect to socio-economic indicators it has been recognized that children's oral health is related to their families' socio-economic status (SES) and their mothers' education level [15-18].

As for behavioural risk factors, human behaviours are often studied through measurement of a person's attitudes. This is based on psychological concepts, which presume that attitudes are relevant determinants of a person's behaviour and vice versa – that behaviour can be predicted from measurable behavioural intentions [19]. One of the theories explaining behavioural patterns is Locus of Control [20], whereby behaviours are being determined by the individual's own ability to control events. A person is determined to be, to varying degrees, a health-external, when one's health is believed to depend on luck, fate or chance, or health-internal, when one believes that health status is determined by one's own behaviour [21]. A more internal locus of control (LoC) is generally seen as desirable, provided it is matched by competence and opportunity so that the person is able to successfully experience the sense of personal control and responsibility [20].

There have been few reports in the literature on the relationship between LoC and caries [22-25]. Furthermore, the findings have been contradictory. The study by Reisine & Litt found that mothers who had more external LoC had children with greater risk of having caries [24], while other studies found no significant differences in either childhood caries [23] or caries relapse [22] between children in different LoC groups. A study on diabetic patients concluded that the ability of psychological characteristics to explain oral health was limited [25]. Only two of those studies analyzed data using regression models including adjustment for potential confounding factors [24,25], with only one study referring to a child population [25]. Therefore, the aim of this cross-sectional survey was to assess the relationship between parental LoC and caries experience and untreated caries of their preschool children in a representative national sample in the Czech Republic, adjusting for relevant sociodemographic characteristics.

## Methods

### Location and study population

**Figure 1:**
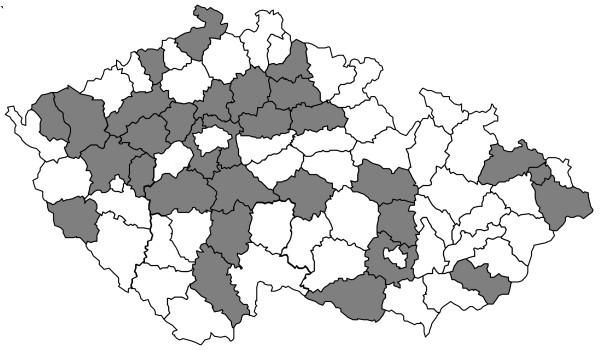
Regions, from which the study sample was recruited (marked in dark colour).

The study was performed in 31 different localities from all regions of the country (see Figure 1). The sites were selected for the purpose of a representative nationwide epidemiological survey by the Czech Institute of Health Information and Statistics. At each site, at least 10 children attending one or two nurseries and aged more than 3 and less than 5 years on the day of the examination were randomly selected. All children were included in the study after signed informed consents of their parents were obtained (a full wording of the informed consent is provided in additional file 1 in doc format). The study has been approved by the Ethical Commission of the General Teaching Hospital in Prague.

At the beginning, 380 families with preschool children were invited to participate in the study, i.e. to sign informed consents concerning the dental examination of their children and the self-completed parental questionnaires. Subsequently, 320 informed consents were received (response rate: 84%). At the same time, 320 self-completed parental questionnaires were also returned. However, at the time of the clinical dental epidemiological examination not all children were present at nurseries; therefore due to missing clinical data, analysis was based on 285 children with completed clinical exams and respective parental questionnaires.

Dental examination of the children was conducted in classrooms, by two calibrated examiners, using dental mirror, rounded probe and headlight. One of the examiners (ZB) had been previously calibrated in a WHO calibration course. The calibration exercise for dental caries was carried out on 30 subjects as recommended by the WHO [26] and inter-examiner agreement was assessed using Cohen's Kappa and considering the entire dentition. A high inter-examiner reliability (Kappa values >0.85) was reached.

### Variables

Caries experience of the primary dentition was clinically recorded in the form of dmft index (d – number of teeth with untreated dental caries, m – number of teeth extracted due to dental caries, f – number of teeth with caries treated with a filling or crown). Level of untreated caries was labelled dt (number of teeth with untreated dental caries). Dental caries diagnostic criteria recorded the disease for distinct to extensive cavity with visible dentine (codes 5 and 6 according to International Caries Detection and Assessment System, ICDAS II) [27]. As a rule, the examiners assigned a lower score when in doubt about the presence/severity of the carious evolvement.

Parents of the examined children were asked to complete a questionnaire based on psychological theoretical models presenting personal attitudes and beliefs as behaviours' predictors. Each parental questionnaire contained information on the child's age and sex, attitudinal items associated with parental oral health beliefs related to dental decay and information on the sociodemographic background of the family (parent's age, sex, marital status and education of the child's mother).

The LoC questionnaire items were taken from a standardized and validated questionnaire, which was employed in an international study on childhood caries [14]. Based on the Locus of Control theory, 13 items that were closely related to the relevant concept were selected (see Table 2). Statements expressing internal LoC were the following: 1, 2, 5, 7, and 11. External LoC is represented by the following items: 3R, 9R and 13R, while beliefs in bad luck/chance are reflected in items: 4R, 6R, 8R, 10R and 12R. Each item was measured on a five-point Likert multi-item scale (ranging from strongly disagree (1) to strongly agree (5)). The coding for the negatively formulated items (items expressing more external LoC or relying on chance) was reversed so that for all items higher score reflected more positive attitudes (stronger internal LoC). Missing data was imputed, allowing for up to 3 missing values.

Parental age was recorded in three categories: "up to 30 years of age", "31–40 years of age" and "above 40 years"; marital status was recorded in two categories: "married" and "single, divorced/separated or widowed" and education of the child's mother was recorded in three categories: "primary school or no formal education", "secondary school" and "further education (college) or higher education (university)".

### Data analysis

Attitudinal items based on LoC theory were subjected to reliability analysis to test the internal consistency of the data. From the LoC attitudinal items, an aggregated Likert-scale score was calculated for each individual respondent, and then the aggregate scores for the whole sample were sorted in increasing order and divided into quintiles, thus producing five categories of LoC ranging from the weakest to the strongest LoC. The quintiles were used in the subsequent regression analyses.

The association between parental LoC and children's caries experience (dmft) and level of untreated caries (dt) was analysed using logistic regression. The outcome variables referred to dental caries measures. Regression analyses were performed for the dmft as the dependent variable and subsequently the same analyses were repeated for dt as the dependent variable. In both cases, the reference category of the dependent variable included children with caries; dmft>0 and dt>0 respectively. First, crude odds ratios (OR) and 95% confidence intervals (95% CI) were presented for the caries experience in the LoC quintiles. These were subsequently adjusted for the effect of child's age and sex, and in a third stage for the effect of child's age and sex, parental age and marital status and education of mother. For statistical analysis of the data, STATA 9 (Stata Corp, College Station, Texas, USA) was used.

## Results

Table 1 shows the descriptive characteristics of the study sample. The study sample consisted of 159 boys (55.8%) and 126 girls (44.2%). The mean age of the children was 4.3 years (ranging from 3.6 to 4.8 years). In terms of dmft, the children were divided into two groups – 146 children (51.2%) without caries experience (so-called intact children; dmft=0) and 139 children (48.8%) with at least one tooth with dental decay, filling or missing due to caries (dmft>0). In terms of untreated caries (dt), there were 162 children (56.8%) with no tooth with untreated caries (dt=0) and 123 children (43.2%) with at least one tooth with untreated caries (dt>0). Almost half of the parents were aged less than 30 years and half aged 31–40 years. More than 80% of the parents were married (indicating complete families). Approximately two thirds of the mothers (64.2%) had secondary education, and approximately one quarter (27.4%) had college or university education.

**Table 1:** Description of the study sample

**Variable**		**Mean (SD)**
Child's age	Years	4.3 (0.5)
	**Category**	**N (%)**
Child's sex	Boys	159 (55.8%)
	Girls	126 (44.2%)
Child's caries experience	dmft = 0	146 (51.2%)
	dmft>0	139 (48.8%)
	dt = 0	162 (56.8%)
	dt>0	123 (43.2%)
Parental age	<30 years	130 (45.6%)
	31–40 years	141 (49.5%)
	>40 years	11 (3.9%)
	Missing data	3 (1.0%)
Parental marital status	Married	235 (82.5%)
	Single/divorced/separated or widowed	50 (17.5%)
Education of the child's mother	Primary school or no formal education	18 (6.3%)
	Secondary school	183 (64.2%)
	College or university	78 (27.4%)
	Missing data	6 (2.1%)

The frequency distribution of attitudinal items is shown in Table 2. In most of the items positive attitudes reflecting tendency to internal control of the disease (scores 4 and 5 after the recoding of items) outbalanced undecided and negative responses. Cumulative frequencies of positive responses ranged from 55.0% for the statement 4: "No matter what we do, our child is likely to get tooth decay" to 93.6% for the statement 2: "As parents, it is our responsibility to prevent our child getting tooth decay", while frequencies of undecided ranged from 6.0% for the statement 2 to 28.4 for the statement 4 and negative responses from 0.4% for the statement 2 to 16.6% for the statement 4. The two exceptions were the statements 10: "Some people just naturally have soft teeth" and 13: "The dentist is the best person to prevent tooth decay in our child", where parents remained generally undecided (42.2% and 31.2% respectively) and also reflected a tendency to fatalism (statement 10, 45.4%) or external control provided by the dentist (statement 13, 34.8%).

**Table 2:** Distribution of individual items of Locus of control (LoC)

**13 attitudinal items** **(285 cases)**	**Possible answers (codes)**			**Mean** **(SD)**
	
	**Agree** **(1–2)**	**Do not know (3)**	**Disagree (4–5)**	
	**%**	**%**	**%**	

1. As a family, we are confident that we can reduce the chances of our child getting tooth decay.	1.4	10.5	88.1	4.04 (0.57)
2. As parents, it is our responsibility to prevent our child getting tooth decay.	0.4	6.0	93.6	4.23 (0.58)
3R. It is the responsibility of the dentist to prevent our child getting tooth decay.	16.5	22.9	60.6	2.46 (0.84)
4R. No matter what we do, our child is likely to get tooth decay.	16.6	28.4	55.0	2.45 (0.93)
5. We can prevent tooth decay in our child by reducing sugary foods and drinks between meals.	5.3	15.3	79.4	3.91 (0.74)
6R. It just happens that children get tooth decay.	16.4	13.2	70.4	2.20 (1.04)
7. If we brush our child's teeth twice a day, we can prevent our child getting tooth decay in the future.	6.0	27.5	66.5	3.74 (0.76)
8R. If our child gets tooth decay, it is by chance.	6.5	26.5	67.0	2.26 (0.75)
9R. It would not make any difference to our child getting tooth decay, if we helped him/her brush every day.	9.7	26.3	64.0	2.36 (0.81)
10R. Some people just naturally have soft teeth.	45.4	42.2	12.4	3.33 (0.80)
11. As a family, we intend controlling how often our child has sugary foods or drinks between meals.	4.9	21.5	73.6	3.77 (0.67)
12R. It is just bad luck if our child gets tooth decay.	13.3	28.3	58.4	2.44 (0.90)
13R. The dentist is the best person to prevent tooth decay in our child.	34.8	31.2	34.0	3.03 (0.92)

* items labelled R were in negative format and were reversed before the analysis

The Cronbach's standardised alpha of the LoC scale was 0.80, clearly demonstrating the successful internal consistency reliability of the attitudinal data. This provides support for the use of the aggregate LoC scale. Aggregated Likert-scale scores of individual respondents ranged from 34 to 62, and the ranges of individual quintiles were as follows: 1^st ^quintile: 34–42; 2^nd ^quintile: 42.25–45.5, 3^rd ^quintile: 46–48.1, 4^th ^quintile: 48.8–51, 5^th ^quintile: 52–62.

As seen from the results presented in Table 3, the crude association between parental LoC and untreated caries of children showed an increasing linear trend in the odds ratios referring to the chance of the child to be free from untreated caries with stronger parental LoC. This means that children in the highest parental LoC quintile were more likely to be free from untreated caries in comparison to their counterparts in the lower quintiles. The same pattern was observed in both levels of adjustment, i.e. after adjusting for the effect of age and sex of the child and after adjusting for the effect of age and sex of the child, parental age and marital status and education of mother. Therefore, neither level of adjustment had any significant effect on the ORs. After full adjustment, children in the strongest parental LoC quintile were 2.81 (1.23–6.42, p<0.05) times more likely to be free from untreated caries in comparison to children in the weakest parental LoC quintile, while the respective figures for children in the 4^th ^, 3^rd^ and 2^nd ^quintile were 2.68, 2.19 and 1.25 times. When all the quintiles were included in the analysis as a single variable, the linear trend of ORs was highly significant (p=0.005 for crude logistic regression, 0.005 for adjustment for age and sex of the child and 0.003 for full adjustment).

**Table 3:** The effect of Locus of control on dt: OR (95% CI) for being free from untreated carries

**LoC**	**Crude**	**Adjusted for age and sex of child**	**Fully adjusted^1^**
**1^st ^quintile (low) (34–42)**	1	1	1
**2^nd ^quintile (42.1–45.5)**	1.13 (0.54–2.38)	1.11 (0.52–2.36)	1.25 (0.56–2.80)
**3^rd ^quintile (45.6–48.5)**	1.91 (0.92–3.97)	1.90 (0.92–3.95)	2.19 (1.00–4.78)*
**4^th ^quintile (48.6–51)**	2.27 (1.09–4.73)*	2.26 (1.09–4.72)*	2.68 (1.21–5.93)*
**5^th ^quintile (high) (51.1–62)**	2.38 (1.11–5.10)*	2.40 (1.12–5.15)*	2.81 (1.23–6.42)*
***P for linear trend of ORs***	***0.005***	***0.005***	***0.003***

^1 ^adjusted for children's age and sex, parental age and marital status and education of mother

* p < 0.05

When dmft was the dependent variable, it was observed that with increasing parental LoC a chance for their children to have intact teeth increased. Table 4 shows the results from this analysis. In this case, there was an increasing linear trend of odds ratios referring to the chance of the child to have intact teeth up to the 4^th ^parental LoC quintile while in the 5^th ^quintile the OR slightly dropped (but was not lower than the values for the 3^rd ^quintile). The same pattern, i.e. linear trend of increasing ORs up to the 4^th ^quintile with a slight decrease of OR in the 5^th ^quintile was observed after both levels of adjustment. Again, neither level of adjustment had any significant effect on the ORs. After full adjustment, children in the strongest parental LoC quintile were 2.32 (1.02–5.25, p<0.05) times more likely to be free from untreated caries in comparison to the weakest parental LoC quintile, while the respective figures for children in the 4^th^, 3^rd ^and 2^nd ^quintile were 3.21, 2.18 and 1.42 times. When all the quintiles were included in the analysis as a single variable, the linear trend of ORs was highly significant (p=0.007 for crude logistic regression, 0.008 for adjustment for age and sex of the child and 0.008 for full adjustment).

**Table 4:** The effect of Locus of control on dmft: OR (95% CI) for having intact teeth

**LoC**	**Crude**	**Adjusted for children's age and sex**	**Fully adjusted^1^**
**1^st ^quintile (low)**	1	1	1
**2^nd ^quintile**	1.28 (0.60–2.72)	1.29 (0.60–2.77)	1.42 (0.62–3.22)
**3^rd ^quintile**	1.91 (0.92–3.96)	1.91 (0.92–3.98)	2.18 (1.00–4.78)
**4^th ^quintile**	2.78 (1.33–5.82)*	2.80 (1.33–5.86)*	3.21 (1.44–7.15)*
**5^th ^quintile (high)**	2.10 (0.99–4.45)	2.08 (0.98–4.42)	2.32 (1.02–5.25)*
***P for linear trend of ORs***	***0.007***	***0.008***	***0.008***

^1^adjusted for children's age and sex, parental age and marital status and education of mother

* p < 0.05

## Discussion

The main finding of this study is that there was a clear linear association between parental LoC and child's caries status; stronger parental LoC was linked with higher probability of their child being free from untreated dental decay in the primary dentition. This indicates a positive effect of strong parental LoC on the level of untreated caries of their preschool children in the study sample. The same pattern was observed in both levels of adjustment and moreover, the linear trends of ORs for both crude and adjusted analyses were highly statistically significant. After adjusting for the effect of various relevant sociodemographic variables, children whose parents had stronger LoC had an increased chance to be free from untreated caries more than twice when compared to children in the lower parental LoC quintiles. That means that the effect of parental LoC on dt was independent from an extended number of key sociodemographic variables for both children and parents (age and sex of child, parent's age, sex, marital status and education of the child's mother), which are potential confounding factors.

With respect to the children's caries experience (dmft), a similar pattern was observed: increasing trend of ORs referring to the chance of the child to have intact teeth with increasing strength of parental LoC up to the fourth quintile with a slight decrease of OR in the 5^th ^quintile. Again, the same pattern was observed in both levels of adjustment and the linear trends of ORs for both crude and adjusted analyses were highly statistically significant confirming that the effect of parental LoC on caries experience was independent from key sociodemographic variables of children and parents. After the full adjustment, children whose parents were in the strongest (4^th ^and 5^th^) LoC quintiles had a more than twice increased chance to have intact primary teeth in comparison to children in the lower parental LoC quintiles.

Though both aforementioned linear associations were statistically significant, there was a stronger and unquestionable linearity in the association between LoC and dt, while the same was not exactly the case for the association between LoC and dmft, where the trend did not apply to the 4^th ^and 5^th ^quintiles. When trying to explain differences in the linearity pattern between the two associations, it is worth considering that dt is a measure of current untreated disease and therefore it is expected to relate in a more linear fashion with LoC, while dmft is a measure of disease experience (both treated and untreated) and is also influenced by treatment provision. Parents with the strongest LoC may also enhance provision of necessary dental treatment to the children. This, in turn, leads to higher dmft through an increase in the number of filled teeth, while having no (or very few) teeth with untreated dental decay. On the other hand, parents with lower LoC may not equally facilitate dental treatment provision and their children's dmft is almost entirely a reflection of untreated dental decay. Therefore, differences in the prevalence of dt between different parental LoC groups may be partially masked when comparing dmft (i.e. caries experience) due to the effect of treatment provision particularly in the higher parental LoC groups, though the very low prevalence of filled teeth in this sample did not allow for further exploring this issue.

The study caries diagnosis threshold was code 5 and 6 according ICDAS II. These codes correspond to obvious cavities, i.e. severe caries. Grades 3–4 of ICDAS II refer to the localized enamel breakdown and underlying dark shadow from dentine, but the diagnosis of these caries stages might have been complicated as the examination was performed under epidemiological field conditions. This means that the study measured the prevalence of cavitated caries lesions in an epidemiological setting rather than total caries experience in a clinical setting. The diagnostic criteria were uniform across the sample and therefore should not have had any influence in the relationship between LoC and untreated caries. The same is not necessarily the case for ft, and hence dmft, which do not assess disease per se but are also influenced by treatment decisions and there is the possibility that some teeth might have been filled while classified in the Grades 3–4 of the ICDAS II system, with others might have been filled while suffering from severe caries (Grades 5–6 of ICDAS II classification). Obviously, this hypothetical lack of homogeneity in the ft component could not have been assessed in a cross-sectional study. However, in any case, it should have no measurable effect on our results due to the very low prevalence of filled teeth in this population.

Looking into the potential role that environmental issues that may have played in influencing the observed family decisions, it should be noted that in the Czech Republic dental insurance is included in general health insurance, which is compulsory for the whole population. In subjects with no income (such as children, unemployed, retired etc.) the medical insurance is paid by the state. Therefore, theoretically there are no policy restrictions in the children's access to dental care. The paediatricians inform all mothers about the obligation to bring the child for the first dental check-up shortly after the eruption of the first tooth. However, as this is left entirely to the responsibility of the parents without any incentives or penalties (e.g. dental insurance companies do not monitor the periodicity of the preventive dental checkups of the insured subjects), it is common practice for parents to bring their child to the dentist for the first time when the child has dental pain or another major dental problem. Currently only a few optional projects on oral health are being carried out at some Czech nurseries (e.g. healthy smile etc.). In relation to the potential effect of fluorides in the Czech Republic, the municipal water is not fluoridated, the vast majority of the children's toothpastes available on the market contain from 500 to 1000 ppm of fluoride, and there are no obligatory official guidelines on the systemic administration of fluorides. It is therefore assumed that the environmental background of the examined children and their families was generally similar.

The findings of this study support the hypothesis that a strong internal parental LoC indicates better control of caries in their children and show that a more internal LoC is desirable. A positive influence of strong parental LoC on children's caries experience was rather expected. But in addition to that, the results showed a statistically highly significant gradual trend between increasing parental LoC and increasing chance of the children to be free from untreated caries and to have intact teeth after adjusting for the effect of for age and sex of the child, parental age and marital status and education of the mother.

Our findings are contradictory to previous studies, which found no significant association between LoC and either childhood caries [23] or caries relapse [22]. However, these studies have not attempted to adjust the association for the effect of potential confounders. On the other hand, our results are in line with those reported by Reisine & Litt in Connecticut, who found that mothers who had more external LoC had children with greater risk of having caries after controlling for the effect of confounders [24]. A study in Finland that also allowed for adjustment of the results for potential confounders concluded that the ability of psychological characteristics to explain oral health was limited [25]; however, as it referred to a mostly adult sample of insulin-dependent diabetic patients, its comparability with our study is rather limited.

This is the first study investigating the role of parental LoC in the caries of preschool children in Central Europe. Furthermore, it was based on a nationally representative, though not very big, sample. The children have been included in the study based on signed parental informed consent. Therefore, the results may not fully reflect the situation in the whole Czech population, especially in relation to the high proportion of children with University educated mothers. Parents of children with extremely poor oral health may often refuse consent to the dental examination, possibly because they are concerned about their neglect to care for their children's teeth being disclosed. Furthermore, in self-reported questionnaire data there is generally the risk that the respondents report what they perceive as the correct answer rather than what they actually believe or do. Finally, assessing the effect of parental LoC on children's caries prevalence and experience requires going beyond the current cross-sectional study into employing a longitudinal design.

## Conclusion

The study findings support the hypothesis that higher internal parental LoC is associated with better control of both untreated caries and caries experience of their children. There was a statistically highly significant linear trend between increased parental LoC and higher probability of the children to be free from untreated caries, independent from the effect of sociodemographic variables of children and parents. A similar highly statistically significant trend, although not entirely linear, and independent from sociodemographic variables was observed with respect to the chance of the children to be free from caries experience with increasing strength of parental LoC. Children of parents with the strongest LoC were more than twice more likely to be free from untreated caries and to have intact teeth in comparison to the children of parents with the weakest LoC, after adjusting for the effect of age and sex of the child, parental age and marital status and education of the mother.

## Competing interests

The authors declare that they have no competing interests.

## Authors' contributions

ZB and EL conceived of the study and collected and processed the data. HP performed the statistical analysis. GT assisted with the strategy for analysis and data interpretation. All authors read and approved the final manuscript.

## Pre-publication history

The pre-publication history for this paper can be accessed here:



## Supplementary Material

Additional file 1 Informed consent. A full wording of the informed consent. Click here for file
