# Supplementary material for: Relationship between parental locus of control and caries experience in preschool children – cross-sectional survey
Source: BMC Public Health. 2008 Jun 12;8:208. doi: 10.1186/1471-2458-8-208 (PMC2442069; doi:10.1186/1471-2458-8-208)
Supplement: Additional file 1 — Informed consent. A full wording of the informed consent. [file 1471-2458-8-208-S1.doc]

**Information Sheet for Parents/Guardians**

**Invitation to join the study**

*We would like to ask you to take part in a scientific study, which aims to provide a better understanding of why some children develop tooth decay. Before you decide whether or not to participate in the study, you need to understand what research is being carried out and what it will involve. Please read the following information carefully. If there is anything unclear, or if you need more information, please do not hesitate to contact us. Thank you for your time.*

**What is the purpose of the study?**

The purpose of this study is to better understand why some children develop tooth decay and others do not. We know quite a lot about tooth decay these days. In this study, we are interested in so called minor risk factors for dental decay (such as your health attitudes and beliefs), which themselves do not directly cause dental decay, but could play role in the process of the disease development.

**Why have I been chosen?**

You have been chosen because you live in one of the sites, which have been chosen for the study and you are the parent/guardian of a child aged 3 to 5 years. We expect to involve 380 families (parents/guardians and children) in this study.

**Do I have to participate?**

It is up to you to decide whether or not to participate. If you do decide to take part you will be given this information sheet to keep and be asked to sign an informed consent form. Even if you decide to take part you are free to withdraw at any time without any reason. Participating in the study will not have any effect on your dental care.

**What will happen if I participate?**

If you agree to participate, we will ask you to answer a questionnaire on dental health and related topics. In the questionnaire you are free not to answer any question without giving any reason.

**What will happen if my child participates?**

If you agree for your child to participate, a qualified dentist will carry out simple and quick dental examination of your child with dental mirror and rounded probe to record the number and condition of your child’s teeth. No additional examination will be carried out and no dental treatment will be provided.

**Are there any risks of participating in the study?**

The risks of participating in the study are the same as having a dental check-up. This is a recognized health practice and to minimize any risks, maximum infection control procedures are being applied. We may find that your child has tooth decay. In such case we will inform you.

**What are the possible benefits of taking part?**

There is no direct benefit to you or your child. However, we hope that our study will provide more knowledge of why some children develop tooth decay.

**Will my taking part in this study be kept confidential?**

All the information, which is collected during the research, will be kept strictly confidential. Any information about study participants will have the names and addresses removed. Each person taking part in the study will be labelled with a unique study number.

**Who is organizing and funding the research?**

The study is organized by experienced research fellows from the Institute of Dental Research in Prague. The study is funded by the Grant Agency of the Ministry of Health of the Czech Republic.

**Contact for further information**

For further information, please contact Dr Zdenek Broukal or Dr. Erika Lencova from the Institute of Dental Research in Prague (tel. +420 242 57 174).

**ID No:**

**CONSENT FORM**

**(Parent/Guardian)**

**Project Title: Oral Health of Preschool Children and Attitudes and Behaviour of Parents in Relation to Prevention of Dental Decay in Primary Dentition**

**Researchers**: **Dr Zdenek Broukal, Dr. Erika Lencova**

1. I hereby confirm that I have read and understood the information sheet related to the above-mentioned study and I have been given opportunity to ask questions.

1. I understand that our participation is voluntary and that we are free to withdraw at any time without giving any reason and without my child’s dental care or legal rights being affected.

3. I agree that my child…………………………………………………(child’s name) and I will participate in the above-mentioned study.

________________________ ________________ ____________________

Name of child’s parent/guardian Date Signature

_________________________ ________________ ____________________

Person taking the consent Date Signature

(If different from researcher)

_________________________ ________________ ____________________

Researcher Date Signature
